# Magna Est Veritas, Et Prævalebit

**Published:** 1881-07

**Authors:** Ad. Fellger

**Affiliations:** Philadelphia


### MAGNUS EST VERITAS, ET PRÆVALEBIT.

AD. FELLGER, M. D., PHILADELPHIA.

THROUGH the kind offices of Prof. Gustav Jæger of Stuttgart, we received in December, 1880, from Herr Hipp, in New Chatel, his well-known chronoscope, which instrument Prof. Jæger used when he instituted his neural-analytical experiments. At the same time we received the first impression of Prof. Jæger's pamphlet on neural-analysis, especially showing its application to the homœopathic potencies.* The efficacy of these potencies has been denied by many heretofore, although thousands and thousands of times has it been proved by actual experiment that these highly potentized homœopathic medicines were curative agents. Now we have obtained proof of their action amounting to an exact certainty, just as exact as the proof of the mechanics of heat by Robert von Mayers; or as that of spectral analysis by Krichoff and Prunsen; or as that of the fourth condition of matter by Crookes. It will be impossible hereafter to find a scientific man bold enough to deny the

* The results of the experiments will be communicated hereafter.

efficacy of these highly potentized drugs, without running the risk of being accused of "ignorance."

Time, the great vindicator of truth, has, finally after the lapse of almost a century, proved the correctness of one of Hahnemann's teachings based on his experiments; and in like manner, time and progress in science will, eventually, just as exactly, prove the correctness of all the other truths he taught, notwithstanding all the opposition by men who refuse obstinately to investigate his teachings by the only possible rational means—experiment.

Everybody is familiar with the absolute laws governing electricity, galvanism and the magnet, *i. e.*, similar poles repel one another, and dissimilar poles attract one another. These laws govern the effect of the magnetic needle—these invisible powers (these homœopathic nothings) of the earth's magnetism, give the north-pole of the magnetic needle its certain directions, thereby becoming an unerring guide to the sea-faring man, to the civil engineer, and to the miner, leading them with a mathematical certainty. This law of polarity is a law governing the universe, and nowhere in nature can a chemical change, or organization on life exist without it.

Hahnemann's fundamental principle, "*similia similibus curantur*," *i. e.*, one similar repels another similar, is precisely the same law, only it is applied to the living organism of men. Physiology teaches that the brain and its ganglia form batteries in which the white substance acts the part of an alkali, and the gray substance an acid (like the two magnetic poles,) and that the nerves act as conductors, and by this very teaching in fact has that science really and truly acknowledged our homœopathic formula *similia similibus curantur* for it would be absolutely impossible to conceive a battery or its action without this law of polarity.

We predict with certainty, that sooner or later physiology will be compelled by necessity to accept this fundamental law; and just as long as she neglects to do so, will she remain barren as a mother to therapeutics. She is also criminally guilty by withholding from the medical world the only compass which may show the physician, as well as the mariner, a safe and speedy way to his destination.

If experimental physiology would accept this principle as a guide to govern its experiments, a conviction would necessarily arise, that out of the acceptance of this principle would follow necessarily the confirmation of all the other principles and rules governing homœopathy, such as the necessity of the small dose, and the necessity of allowing each dose to exhaust its action before administering another one.

Furthermore, will the physiology of the future find a great and unexpected treasure in the homœopathic drug-provings which must lead to new discoveries, showing the connection and reciprocal effects in human organism, which otherwise could never be found in any other way.

The great powers which are developed when electricity or magnetism combines with a substance is exemplified in ozone (electrified oxygen) or in iron, which has been made a magnet by rubbing.

The one thousandth part of ozone in the air is beneficial; one thousandth part more added, kills a great number of small animals; in still larger quantities it suffocates by its effects on the lungs, be they ever so powerful.

A homœopathic, nothing more or less, and on the one hand death is changed into vitality, or on the other, life is turned into death. Ozone possesses a much greater power to oxydize than oxygen, and can turn pure silver into brown silver; hyperoxide air, which contains in one millions part one part of ozone still retains its peculiar odor.

Nobody doubts that iron magnetized by rubbing, attracts iron or steel, because it can be demonstrated *ad oculos*, notwithstanding these stubborn facts, the means of producing this magnetic power must be counted to be one of the homœopathic nothings, as it can neither be seen or weighed, and is discernable only by its effects.

The effect which the magnet has upon the human organism (proved by Hahnemann homœopathically) will therefore hardly be denied by anybody. If a magnetic bar is broken at the point of indifference (in the middle) into two pieces, the parts do not cease to be magnetic, the broken indifferent or neutral point becomes endowed with magnetism. The north-pole portion becomes a south-pole, and the south-pole part becomes north. The same result will follow every new division, and will continue up to the division of the most minute molecules. The magnet has not become inert by division; it has only been divided into innumerable small magnets, and a free movement of the molecules has been made possible, as is the case in the fourth condition of matter (the radiant state). The manner of preparing homœopathic medicines is analagous to the division of the magnet. Their action is just as certainly a scientific fact. Therefore, they are justified not only on scientific principles, but their action is just as certainly proved by experience as is the action of the magnet.

Since Hahnemann's promulgation of homœopathy, new discoveries in natural science have overthrown many theories and hypotheses of the old school; while on the other hand, these progressive discoveries have in every instance been in harmony with homœopathy, and have explained, clearly the correctness and truth of its principles and teachings. The next generation educated in the newly acquired scientific progress will do justice to homœopathy and to homœopathic therapeutics and assign to it the higher scientific position which is due it—a position far above that of the old school. Homœopathy has saved the healing art from the humiliating declaration that the mechanic arts have understood better how to utilize new scientific discoveries—the discoveries of the invisible powers (utilized in the telegraph, the telephone, the galvano-plastic process, the electric light, etc.,) for the benefit of mankind, than the so-called scientific medical men. The contempt which these men have shown to the homœopathic principles and the powerful remedies which they have termed *nothings* will rebound upon them doubly severely, and the ignorance which they exhibited will be so much the longer remembered. This is the lesson history teaches. The natural sciences have arrived upon the threshold of a new era, and we have learned to calculate with smaller potencies than formerly was deemed possible, and the time has come when ideas, the fruits of antiquated reasoning, should be set aside and new ones in harmony with new discoveries be accepted. It shall be my aim to show in future, in another paper how, as above indicated, homœopathy alone remains in harmony with the progressive sciences. Microscopic anatomy, experimental physiology and pathological observation offer us a large amount of material on which we may base our arguments proving our propositions.

